# ECM Protein CYR61 Promotes Migration and Osteoblastic Differentiation of Irradiation BMSCs via Migrasomes

**DOI:** 10.1155/sci/8825935

**Published:** 2025-09-21

**Authors:** Chaoting Yan, Wen Sun, Zhi Chen, Liu Liu, Pin Zhou, Yueguang Gu, Geng Wu, Kunpeng Wang

**Affiliations:** ^1^Department of Stomatology, Lianyungang Clinical College of Nanjing Medical University, The First Affiliated Hospital of Kangda College of Nanjing Medical University, The Affiliated Lianyungang Hospital of Xuzhou Medical University, Lianyungang, Jiangsu, China; ^2^Precision Medicine Laboratory, Lianyungang Clinical College of Nanjing Medical University, The First Affiliated Hospital of Kangda College of Nanjing Medical University, The Affiliated Lianyungang Hospital of Xuzhou Medical University, Lianyungang, Jiangsu, China; ^3^Department of Central Laboratory, Lianyungang Clinical College of Nanjing Medical University, The First Affiliated Hospital of Kangda College of Nanjing Medical University, The Affiliated Lianyungang Hospital of Xuzhou Medical University, Lianyungang, Jiangsu, China; ^4^Department of Endodontics, Jiangsu Province Key Laboratory of Oral Diseases, Jiangsu Province Engineering Research Center of Stomatological Translational Medicine, The Affiliated Stomatological Hospital of Nanjing Medical University, Nanjing, Jiangsu, China; ^5^Surgical Department, Lianyungang Clinical College of Nanjing Medical University, The First Affiliated Hospital of Kangda College of Nanjing Medical University, The Affiliated Lianyungang Hospital of Xuzhou Medical University, Lianyungang, Jiangsu, China

**Keywords:** BMSCs, migrasomes, migration, osteoblastic differentiation, osteoradionecrosis of the jaw

## Abstract

Osteoradionecrosis of the jaw (ORNJ) is a complication of radiation therapy that can lead to hard-to-repair bone defects. Bone marrow mesenchymal stem cells (BMSCs) have been identified as potential “seeds” for restoring bone defects. In this study, we reported extracellular matrix protein cysteine-rich angiogenic inducer 61 (CYR61) to enhance the migratory and osteogenic functions of irradiated BMSCs (IR BMSCs) by migrasomes. Various assays, including alkaline phosphatase (ALP) activity assay, Cell Counting Kit-8 (CCK-8), apoptosis analysis, qRT-PCR, western blot, ALP staining, alizarin red S (ARS) staining, wound healing assay, transwell assay, and co-immunoprecipitation (co-IP) were conducted to assess the optimal radiation dose for generating IR BMSCs and migrasome functionality. Proteomics, bioinformatics analysis, gene transfection, and molecular docking were employed to identify key molecules mediating migration and osteoblastic differentiation and its downstream mechanisms. Furthermore, confocal microscopy, transmission electron microscopy (TEM), and western blot were utilized to identify migrasomes. Results showed that a radiation dose of 2 Gy inhibited migratory and osteogenic abilities of cells without significantly affecting viability. CYR61 emerged as a pivotal molecule regulating BMSC migration and osteoblastic differentiation through binding to integrin αvβ3 at the 125th aspartic acid and activating the ERK signaling pathway. We discovered that migrasomes are the key vehicle effectively delivering CYR61 to restore migration and osteogenesis of IR BMSCs. In conclusion, migrasomes-secreted CYR61 facilitating a promotional effect can regulate the migration and osteogenesis of IR BMSCs. Thus, migrasomes-origin CYR61 may serve as potential therapeutic agents for repairing ORNJ-related bone defects.

## 1. Introduction

Osteoradionecrosis of the jaws (ORNJs) is a complication arising from radiation therapy for head and neck malignancies, often resulting in severe bone defect [1]. Despite advances in radiotherapy and preventive medicine, the incidence of ORNJ remains at approximately 7.52%, with the issue of severe and hard-to-repair bone defects continuing to be a major challenge [2]. In some cases, even with medical costs reaching up to $340,000, patients with severe ORNJ may not achieve satisfactory outcome after conservative and surgical treatment [3]. Therefore, it is crucial to explore more effective methods for repairing bone defects associated with ORNJ.

Bone marrow mesenchymal stem cells (BMSCs) are vital for bone regeneration following radiative injuries and are a primary cell type involved in ORNJ [1]. Recent studies have focused on irradiated BMSCs (IR BMSCs) to understand the causes and potential solutions for this abnormality. They IR BMSCs directly in vitro or extract the IR BMSCs following irradiating their donors to simulate ORNJ and have investigated various factors such as viability, apoptosis, DNA damage, and osteogenic potential for these IR BMSCs [4, 5]. Irradiation with 2 Gy decreased osteoblast mineralization, leading to local bone loss in mice and inhibited osteogenic differentiation in rats [6, 7]. In the osteoblast cell line OCT-1, irradiation (2 Gy) induced a stringent G2-arrest at 8 h postirradiation, which resolved by 24 h. By 48 h, a considerable fraction of cells appeared to resume cycling. However, doses between 2 and 7 Gy induced a dose- and time-dependent cell cycle arrest [8]. In contrast, 0.5 Gy γ-irradiation led to decreased proliferation and osteogenic ability in rat BMSCs [9]. These discrepancies may arise from differences in cell types/models, irradiation doses, and time points analyzed [6, 10].

To our knowledge, there are few reports on migrative inhibition, for instance, a 6 Gy dose has been shown to inhibit the migration of IR BMSCs derived from mice [5]. Given the importance of migration for BMSCs' ability to home and promote regeneration, we aimed to explore this less-studied aspect of IR BMSCs' migratory potential and assess their osteoblastic differentiation, which is also vital for the repair of bone defects [11].

In this study, we have explored that cysteine-rich angiogenic inducer 61 (CYR61) or cellular communication network factor family member 1 (CCN1) is decreased in IR BMSCs by proteomics study. We also investigated that the deficiency of CYR61 result in inhibiting the migration and osteoblastic differentiation of BMSCs and the downstream molecular mechanisms. Our findings revealed that CYR61 in migrasomes derived from BMSCs are promising for treating ORNJ-related bone defects. CYR61, an extracellular matrix protein vital for migration is known to activate the migration-associated protein RhoA [12, 13]. Additionally, CYR61 has been shown to mitigate the decline in osteoblastic potential associated with aging [14]. Furthermore, CYR61 has been demonstrated to directly bind to integrin αv, thereby regulating fibroblast adhesion [15].

Migration is a multifaceted process initiated by adhesion, where cells move towards an injury site [11, 16]. This involves steps such as cell adhesion, the integration of cytoplasmic protrusions, traction, and attachment leading to the reassembly of the cell skeletal [17]. During migration, cells release pomegranate-like vesicles, called migrasomes, with diameters ranging from 0.5 to 3 µm through migracytosis [18]. Migrasomes are newly found organelles that typically attach to retraction fibers (RFs) and are released once these fibers dissipate [18]. They have been shown to enhance cellular movement, contribute to organ morphogenesis, and play roles in tumor development and immune evasion [19, 20]. Owing to their origin, BMSC-derived migrasomes are postulated to effectively promote osteogenesis. Furthermore, BMSCs' migrasomes have demonstrated effectiveness in regulating the immune system and reducing inflammation associated with poststroke pneumonia [21, 22]. Migrasomes influence target cells by delivering mRNA and proteins, with proteins playing a crucial role in their biogenesis and function [23, 24]. For example, biomarkers like tetraspanin 4 (TSPAN4) and EGF domain-specific O-linked N-acetylglucosamine transferase (EOGT) have been identified using techniques such as mass spectrometry [25]. Based on these findings, it is speculated that migrasomes could play a role in transportting key protein molecules to regulate BMSCs' fate within an inflammatory micro environment.

Although extracellular vesicles (EVs) have been linked to ORNJ, their therapeutic effects on ORNJ have not been clearly reported [26]. This study represents the first attempt to use CYR61 as the new target with the new find EVs-like organelle migrasomes as vehicle to enhance both the migration and osteogenic capacity of IR BMSCs, thereby offering a novel strategy for the repair of ORNJ-related bone defects.

## 2. Materials and Methods

### 2.1. Cell Culture, Characteristics, and Reagents

This study involving human specimens was approved by the Ethics Committee Department of The First People's Hospital of Lianyungang City (Approval number: KY-20240407001-01). All study procedures, including notifications and obtaining consent, were conducted in accordance with relevant guidelines. BMSCs were isolated from the jaw bone tissue of patients between 18 and 35 years old who underwent extraction of impacted teeth. The inclusion criteria and cell isolation protocols were conducted as previously described, with BMSCs being used for the following experiments before Passage 15 [27, 28]. The complete medium consisted of DMEM-low glucose (KGM31600-500, KeyGEN BioTECH, China), 10% fetal bovine serum (FBS; c04001, VivaCell BIOSCIENCES, USA), and 1% antibiotic (100 U/mL penicillin and 100 g/mL streptomycin, VC2003, Vicmed, China) [28].

To test osteogenic differentiation, BMSCs were cultured in osteogenic induction medium (OIM), containing 10^−7^ M dexamethasone (CAS:50-0-2, MACKLIN, China), 10 mM β-glycerophosphate (CAS:591-59-3, MACKLIN, China), and 50 μM ascorbic acid (CAS:50-81-7, MACKLIN, China). For adipogenic differentiation induction, BMSCs were cultured alternately in complete medium containing 1 μM dexamethasone, 10 mg/L of insulin (CAS:11070-73-8, MACKLIN, China), 0.2 mM indomethacin (CAS:53-86-1, MACKLIN, China), and 500 μM 3-isobutyl-1-methylxanthine (CAS:28822-58-4, MACKLIN, China) as well as complete medium containing 10 mg/L of insulin, as previously described. Oil Red O (G1262, Solarbio, China) staining was utilized to assess the potential for adipogenic differentiation. For the identification of surface markers using flow cytometric analysis, BMSCs collected with EDTA-free trypsin were separately incubated with CD45, CD29, and CD90 (560975, 561794, and 561969, BD, USA).

To investigate the requirement of ERK in CYR61 function, 10 μM CYR61 (HY-P7842, MCE, USA) in serum-free medium for 24 h and 10 μM specific ERK inhibitor U0126 (HY-12031A, MCE, USA) for 1 h were employed [29].

### 2.2. Generation of IR-Induced BMSCs

The cells were cultured in T25 flasks, and when they reached a confluency of 90%, they were exposed to a single dose of X-ray irradiation from the linear accelerator, as previously described [30]. According to the experimental groups, cells were irradiated with doses of 2, 4, and 6 Gy at a rate of 300 cGy/min in a 15 cm × 15 cm field by medical linear accelerator (Unique, Varian, USA). Untreated cells were included as the control group, following procedures outlined in published literature [31].

### 2.3. Assays for Detecting the Cell Condition

The cell viability was assessed using the Cell Counting Kit-8 (CCK-8) assay following the manufacturer's instructions provided with the CCK-8 kit (C6005, NCM Biotech, China). Specifically, cells were seeded into a 96-well plate. Upon adherence to the plate, they were serum-starved for 24 h prior to the addition of 100 µL of basal medium containing 10 µL of CCK-8. Apoptosis analysis was conducted using flow cytometry after staining the cells with the Annexin V/FITC-propidium iodide (PI) from the Apoptosis Detection kit (KGA105, Kaiji, China). The data were analyzed using FlowJo_V10 software.

### 2.4. Analysis of Cell Mobility

Would healing assay and transwell assay were conducted in this part to detect changes in cell mobility. For the wound healing assay, cells were plated on a six-well plate and allowed to reach confluence of over 90%. A 200 µL pipette tip was then used to create a scratch wound. After 24 h of incubation in serum-free medium, photos of the wound area were collected again to assess cell migration. For the transwell assay, we prepared 8-µm transwell inserts and placed them in a 12-well plate. Then, 1 × 10^4^ cells were seeded on the upper chamber. Serum-free medium was added to the upper chamber, while complete culture medium was added to the lower chambers. After incubating for 24 h, the transwells were washed twice with PBS, fixed with 4% paraformaldehyde, and then, stained with 0.1% crystal violet solution (DZ0055, Leagene, China) for 15 min. Stained cells were photographed using a microscope (XD-101JNOEL, OLYMPUS, Japan).

### 2.5. qRT-PCR Assay

Total RNA was extracted using the Trizol reagent (CWBIO, China). Subsequently, cDNA was synthesized following the instructions provided with the HiScript II RT SuperMix for qPCR kit (R223, Vazyme, China). The primers utilized in this study are listed in Table 1. qRT-PCR was performed on an ABI 7500 FAST Real-Time PCR System using the ChamQ SYBR qPCR Master Mix (Q331, Vazyme, China).

### 2.6. Western Blot Analysis

This assay aimed to assess the expression levels of proteins, including key molecules associated with osteogenesis, migrasome biomarkers, and ERK signaling. The samples were prepared with RIPA (P0013B, Beyotime, China) adding protease inhibitor cocktail (K1007, APExBIO, USA) and phosphatase inhibitor cocktail (K1015, APExBIO, USA). The antibodies used were as follows: runt-related transcription factor-2 (RUNX2; AF5186, Affinity, USA), osterix (OSX; DF7731, Affinity, USA), alkaline phosphatase (ALP; DF6225, Affinity, USA), EOGT (HA721973, HuaBio, China), integrin αvβ3 (ER1911-52, HuaBio, China), ERK1/2 (ET1601-29, HuaBio, China), phospho-ERK1(T202 + Y204) + ERK2(T185 + Y187) (SC58-1, HuaBio, China), TSPAN4 (A10253, ABclonal, China), CYR61 (26689-1-AP, Proteintech, China), β-actin (66009-1, Proteintech, China), HRP conjugated goat anti-rabbit IgG antibody (HA1001, Huabio, China), and HRP-conjugated goat anti-mouse IgG antibody (No. HRP-10283, Proteintech, China). β-actin served as an internal reference for normalization.

### 2.7. ALP Activity Assay

The assay was designed to evaluate the osteoblastic differentiation potential of the cells. After culturing in OIM for 7 days, the cells were gently harvested and ALP activity was assessed using the kit (A059-2, Jiancheng, China) and a microplate reader (iMark, Biorad, USA), measuring absorbance values (OD). These results were normalized by protein concentration, determined using the Bradford kit (P0006C, Beyotime, China).

### 2.8. ALP Staining Assay

This experiment aimed to detect ALP activity more vividly. After induction with OIM for 7 days, the cells were fixed with 4% paraformaldehyde and stained according to the instructions provided with the BCIP/NBT Alkaline Phosphatase Color Development Kit (C3206, Beyotime, China). The photos were then captured using microscopy and a scanner.

### 2.9. Alizarin Red S (ARS) Staining Assay

This assay was designed to demonstrate mineral nodule formation and reflect osteogenic potential. After culturing in OIM for 14 days, fixed cells were stained with ARS staining reagent (C0138, Beyotime, China) for 5 min, and images were captured.

### 2.10. Isolation and Identification of Migrasomes

First, we employed iFluor 488-wheat germ agglutinin (WGA-488; 25530, AAT Bioquest, USA) staining and confocal microscopy to observe migrasomes derived from BMSCs during the migration process. When the cell confluence reached 30%–40%, BMSCs were subjected to serum deprivation for 24 h. Subsequently, both the supernatant and cells were collected and migrasomes were isolated using the previously reported method, which we summarize as differential centrifugation–reverse filtration–ultrafiltration (DC–RF–UF) [32]. The detailed procedure involved sequential centrifugations at 4°C (1000 × *g*, 10 min; 2000 × *g*, 20 min; 8000 rpm, 5 min), retaining the supernatant each time. The final supernatant was passed through a 0.45 µm filter. Migrasomes retained on the membrane were collected by rinsing the filter funnel from the bottom with cold PBS, then concentrated via centrifugation in a 100 kDa UF tube at 2000 × *g* for 5 min. After fixation with an electron microscopy fixative and stained with uranyl acetate and lead citrate as reported in the literature, the appearance of migrasomes was captured using transmission electron microscopy (TEM; HT7800, Hitachi, Japan) [32]. The migrasome biomarkers, TSPAN4, and EOGT, were assessed via western blot analysis in both BMSCs and migrasomes.

### 2.11. Proteomics Study

BMSCs and IR BMSCs, each with three biological replicates, underwent cleavage for proteomic analysis, which was conducted by HOOGEN BIOTECH (Shanghai, China). Following reduction, alkylation, and trypsin hydrolysis, peptides were labeled with tandem-mass-tag (TMT) and subjected to quantitative mass spectrometry. Liquid chromatography-tandem mass spectrometry (LC-MS/MS) was employed for data collection. Protein identification and quantitative analysis were conducted, followed by differential expression analysis and bioinformatics analysis. Pathway analysis was performed using the Kyoto Encyclopedia of Genes and Genomes (KEGG) and Gene Ontology (GO) databases.

### 2.12. Immunofluorescence Staining

Cells were seeded on coverslips and fixed with 4% paraformaldehyde once they reached 30%–40% confluence. They were then permeabilised with 0.5% Triton X-100 and blocked using goat serum. The coverslips were incubated overnight at 4°C with the primary antibody, followed by incubation with either a CY3-conjugated secondary antibody (33108ES60, Yeasen, China) or a FITC-conjugated secondary antibody (33107ES60, Yeasen, China) for 1 h at room temperature. Subsequently, the coverslips were stained with WGA-488 or DAPI solution (BL105A, Biosharp, China). Confocal microscopy was used to examine the localization of CYR61 within migrasomes and its association with integrin αvβ3.

### 2.13. Gene Transfection

Three small interference RNAs (siRNAs) targeting CYR61 were supplied by Genebase (Nanjing, China) to downregulate CYR61 expression. These siRNAs, along with a corresponding control, were transfected into BMSCs using Lipo 8000 (Beyotime, China) according to the manufacturer's instructions. The effectiveness of the transfection was evaluated using qRT-PCR and western blot analyses. Additionally, we also constructed the lentivirus named shCYR61 based on the siRNA with the highest efficiency and pLVX-puro vector from Corues Biotechnology (NanJing, China).

### 2.14. Molecular Docking

To explore the interactions between CYR61 and integrin αvβ3, we performed molecular docking to analyze their potential binding modes. The crystal structures of the proteins were obtained from the Protein Data Bank (PDB). The construction of these structures and the molecular docking processes were predicted using the GRAMM docking web server. These docking results were ranked based on their potential docking probabilities and the top three were further analyzed using PDBePISA. The key binding site in CYR61 was represented by PyMol 3.0.

### 2.15. Co-immunoprecipitation (co-IP)

This assay evaluated the binding of CYR61 and its downstream integrin αvβ3. 10^8^ BMSCs were lysed by cell lysis buffer for western and IP (P70100, NCM, China) adding protease inhibitor cocktail (K4001, ApexBio, USA). We used CYR61 rabbit antibody (ab230947, Abcam, UK) and Protein A/G magnetic beads (HY-K0202, MCE, USA) to precipitate CYR61 and associating molecule. Mouse-origin antibody CYR61 (PTM-6444, PTM BIO, China) and integrin αvβ3 antibody (ER1911-52, HuaBio, China) were applied to analysis corresponding proteins expression by western blot.

### 2.16. Statistical Analysis

Statistical analysis was performed using SPSS 17.0 software. Student's *t*-test was employed to compare differences between two groups, while differences among more than two groups were assessed using ANOVA. A *p*-value of less than 0.05 was considered statistically significant.

## 3. Results

### 3.1. Isolation and Characterization of BMSCs

**Figure 1:**
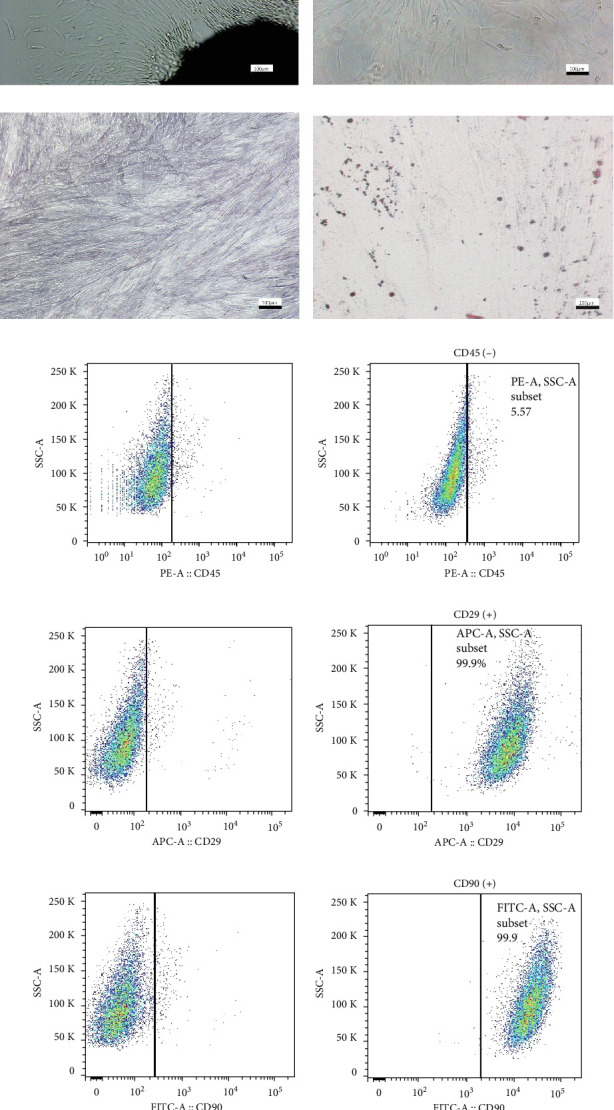
Cell culture and identification. (a) Primary BMSCs. (b) Passage 3 BMSCs. (c) ALP staining of BMSCs following culture in osteogenic induction medium. (d) Oil red O staining of BMSCs after adipogenic induction. (e–g) Examination of the surface markers on BMSCs using flow cytometry, including CD45 (5.57%), CD29 (99.9%), and CD90 (99.9%). The control cells without staining are displayed in the left panel of the charts. Scalar bar = 100 µm.

Primary BMSCs were derived from jaw bone tissue (Figure 1a), and Passage 3 BMSCs maintained an adherent-growth pattern and exhibited a spindle-like shape (Figure 1b). Upon induction with OIM, the majority of BMSCs demonstrated positive ALP staining, confirming their osteoblastic capabilities (Figure 1c). Additionally, after adipogenic induction, lipid droplets stained with oil red O were evident, verifying the adipogenic potential of the BMSCs (Figure 1d). The capability for both osteogenic and adipogenic differentiation indicated the multi-differentiation potential of these stem cells. Surface marker analysis using flow cytometry showed that the BMSCs lacked the hematopoietic marker CD45 and expressed the mesenchymal markers CD29 and CD90 (Figure 1e–g). These findings related to multidifferentiation potential and surface marker expression establish the mesenchymal stem cell identity of the BMSCs.

### 3.2. The Migratory and Osteogenic Differentiation Capabilities of IR BMSCs Were Inhibited

**Figure 2:**
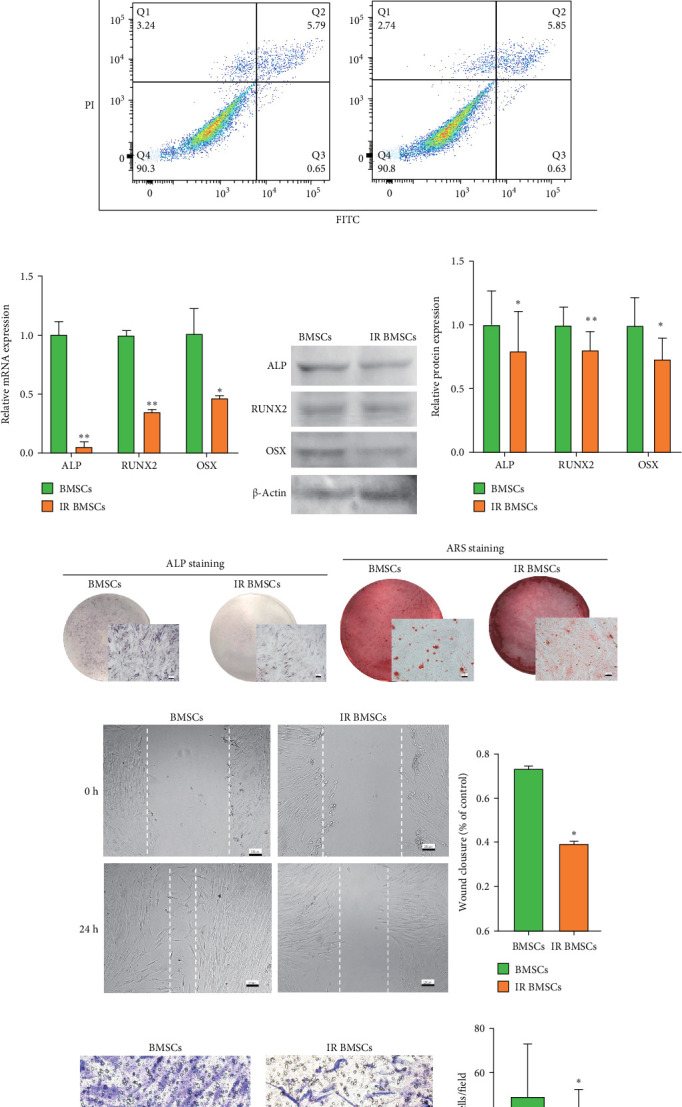
Construction of IR BMSCs and validations for diminished osteogenic and migrative potential of IR BMSCs. (a) Among 2, 4, and 6 Gy, notable changes were observed starting from 2 Gy, as determined by ALP activity assay. (b) CCK-8 assay revealed no significant differences in cell proliferation between BMSCs and IR BMSCs (2 Gy radiation). (c) Flow cytometry analysis of apoptosis levels showed no significant difference between IR BMSCs and its control. (d) Expression of *ALP*, *OSX*, and *RUNX*2. (e) The results of ALP, OSX, and RUNX2 expression. (f) Histograms showed the quantification of band intensities. (g) ALP staining results. (h) ARS staining results. (i) Would healing assay showed IR BMSCs' decreased migrative potential. (j) Transwell assay results. Scalar bar = 100 µm. Data are represented as mean ± SEM, *n* = 3, *⁣*^*∗∗*^*p* < 0.01, *⁣*^*∗*^*p* < 0.05.

The ALP activity assay showed that radiation affected osteogenic differentiation with 2 Gy, which initially demonstrated a less pronounced effect (Figure 2a). There was no significant change in proliferation following exposure to 2 Gy irradiation (Figure 2b). Apoptosis rates, determined through flow cytometry, were similar between IR BMSCs (treated with 2 Gy) and the control group (Figure 2c), leading to the selection of 2 Gy as the radiation dose for further studies. qRT-PCR analysis revealed reduced expression of osteogenesis-associated genes (*ALP*, *OSX*, and *RUNX2*) in IR BMSCs (Figure 2d). This decrease was consistent at the protein level, as shown by western blot analyses of band intensities (Figures 1f and 2e). In the IR BMSCs group, fewer cells were positive for ALP staining (Figure 2g) and there was a decrease in the number of mineralized nodules, indicated by ARS staining (Figure 2h). The wound healing assay showed a smaller closure area in IR BMSCs after 24 h, suggesting reduced cell mobility (Figure 2i). Additionally, the number of transmigrated cells was significantly lower in IR BMSCs compared to control BMSCs (Figure 2j).

### 3.3. ECM Protein CYR61 Was Decreased in IR BMSCs Comparing to BMSCs

**Figure 3:**
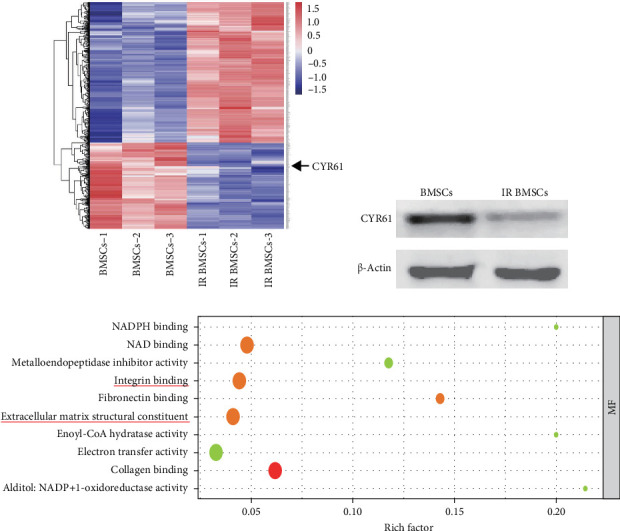
Proteomic technology identified the key molecule influencing the diminished osteogenic and migratory abilities of IR BMSCs. (a) Heat map revealed the differential expression proteins between BMSCs and IR BMSCs. CYR61 was found to be downregulated in IR BMSCs. (b) Western blot verified the proteomics data. (c) GO analysis of analysis on molecular functions suggested the significance of migrasome-related integrin binding (*n* = 3).

To identify the key molecule involved in reducing the mobility and osteogenic properties mediated by irradiation, we carried out a proteomics analysis that led to the identification of 560 proteins (347 upregulated and 213 downregulated) that met our inclusion criteria (*p*  < 0.05, fold change > 1.2 for upregulation, < 0.83 for downregulation). CYR61, among the downregulated proteins, particularly stood out (Figure 3a), a finding further supported by western blot analysis (Figure 3b). GO analysis indicated that CYR61-related functions, such as “integrin binding” and “extracellular matrix structural constituent” were among the top 10 signaling pathways influenced (Figure 3c). As a key cellular growth factor in bone fracture repair, CYR61 belongs to the cellular communication network (CCN) protein family [33]. Members of this family primarily exert their functions via integrin receptor binding during skeletal development, injury repair, and inflammatory processes [34]. Notably, local administration of CYR61 significantly enhances bone strength and endothelial cell adhesion [33].

### 3.4. CYR61 Promoted Migration and Osteogenesis by Binding Integrin αvβ3 via ERK Signaling

**Figure 4:**
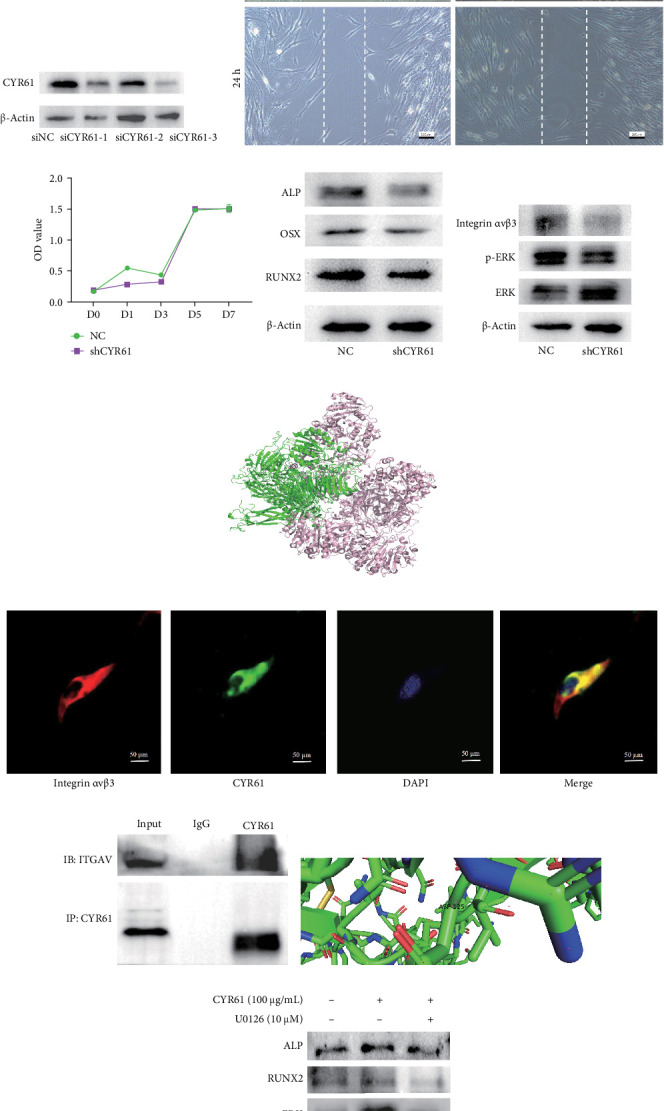
Potential role and molecular mechanism of CYR61 mediate the migration and osteogenesis of IR BMSCs. (a) Sequences of the siRNAs targeting CYR61. (b) Efficacy of CYR61 siRNAs at reducing mRNA levels. (c) Protein levels confirming the inhibitory efficiency of CYR61 siRNAs. (d) Results of the wound healing assay. Scalar bar = 100 µm. (e) Cell proliferation assessed by CCK-8 assay. (f) Western blot analysis showing levels of ALP, OSX, and RUNX2 following CYR61 knockdown. (g) Impact of CYR61 knockdown on integrin αvβ3 and ERK signaling pathway. (h) Results of the molecular docking assay. (i) Immunofluorescence localization of CYR61 and integrin αvβ3. (j) Co-IP analysis demonstrated the specific interaction between CYR61 and integrin αvβ3. (k) Diagram showing the potential binding site of CYR61 with integrin αvβ3. Scalar bar = 50 µm. (l) Rescue assay showing that CYR61-induced increases in osteogenic markers (ALP and RUNX2) and ERK phosphorylation are partially reversed by U0126-mediated ERK inhibition. Western blot detection. (m) Wound healing assay revealing that CYR61-enhanced cell migration is suppressed by U0126 treatment. Data are represented as mean ± SEM, *n* = 3, *⁣*^*∗∗*^*p* < 0.01, *⁣*^*∗*^*p* < 0.05.

To investigate the role of CYR61 in migration and osteogenesis, we constructed three siRNAs (Figure 4a). Based on the qRT-PCR and western blot results, which screened for efficiency, we selected the siRNA labeled “siCYR61-3” for further research (Figure 4b,c). Knockdown of CYR61 led to slower wound healing (Figure 4d). The application of a lentivirus based on siCYR61-3 did not affect BMSC proliferation, as indicated by the CCK-8 assay (Figure 4e), but resulted in a significant decrease in the expression of ALP, OSX, and RUNX2 (Figure 4f). To elucidate CYR61's functional mechanism, we used the STRING website to predict potential downstream targets, focusing on integrin αv (Figure S1a). We found integrin αvβ3, a common dimer including integrin αv, as playing a critical role in regulating migration. Since the interaction between CYR61 and integrin αvβ3 (α subunit) was previous demonstrated by assays in other cell types, we visualizing this relationship through molecular docking (Figure 4h). We observed the coexpression of CYR61 and integrin αvβ3 in the plasma through immunofluorescence staining (Figure 4i). Co-IP was used to assess the direct binding of CYR61 to integrin αvβ3 (Figure 4j). The binding site at the 125th aspartic acid was visualized using PyMOL-3.0.3 (Figure 4k) and the amino acid sequence surrounding this site was found to be highly conserved across various species (Figure S1b). Additionally, we confirmed that the ERK signaling pathway is a downstream mechanism for mediating CYR61's function associating integrin αvβ3 (Figure 4g). Rescue assays showed that U0126-mediated inhibition of ERK signaling abolished the CYR61-induced promotion of cell migration and osteoblastic differentiation (Figures 4l,m).

### 3.5. CYR61 Was in the Migration-Related Vesicles, Known as Migrasomes by BMSCs

**Figure 5:**
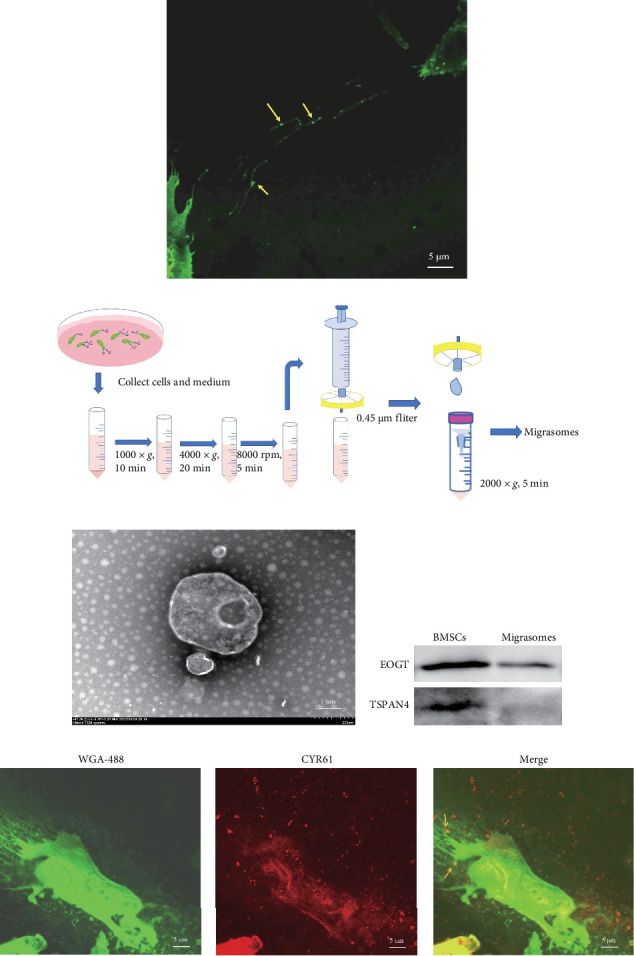
CYR61 could be secreted into the extracellular space via migrasomes. Extraction and characteristics of migrasomes derived from BMSCs. (a) Migrasomes from BMSCs (highlighted by yellow arrows), showing green fluorescence of contractile filaments stained with WGA-488, as observed by laser confocal microscopy. Scalar bar = 5 µm. (b) A schematic representation of the process for extracting migrasomes. (c) Transmission electron microscopy (TEM) captured the appearance of the pomegranate-like vesicles. Scalar bar = 1 µm. (d) Western blot analysis confirmed the presence of specific markers of migrasomes. (e) CYR61 was expressed in both migrasomes and origin cells. Scalar bar = 5 µm. One representative experiment is shown (*n* = 3).

We observed migrasomes (indicated by yellow arrows) and their associated RFs, which emitted green fluorescence under WGA-488 staining (Figure 5a). Since BMSCs are capable of secreting migrasomes, we employed a previously reported method known as DC–RF–UF, illustrated in the flow-process diagram (Figure 5b), for their isolation. TEM provided detailed images of the vesicles, highlighting smaller internal vesicles (Figure 5c). The presence of migrasome biomarkers, EOGT and TSPAN4, was confirmed in both the BMSCs and the migrasomes isolated by us (Figure 5d). Additionally, we confirmed the presence of CYR61 in migrasomes derived from BMSCs through fluorescence localization (Figure 5e).

### 3.6. Migrasomes-Derived From BMSCs Could Supplement With CYR61 and Rescue the Migrative and Osteoblastic Ability of IR BMSCs

**Figure 6:**
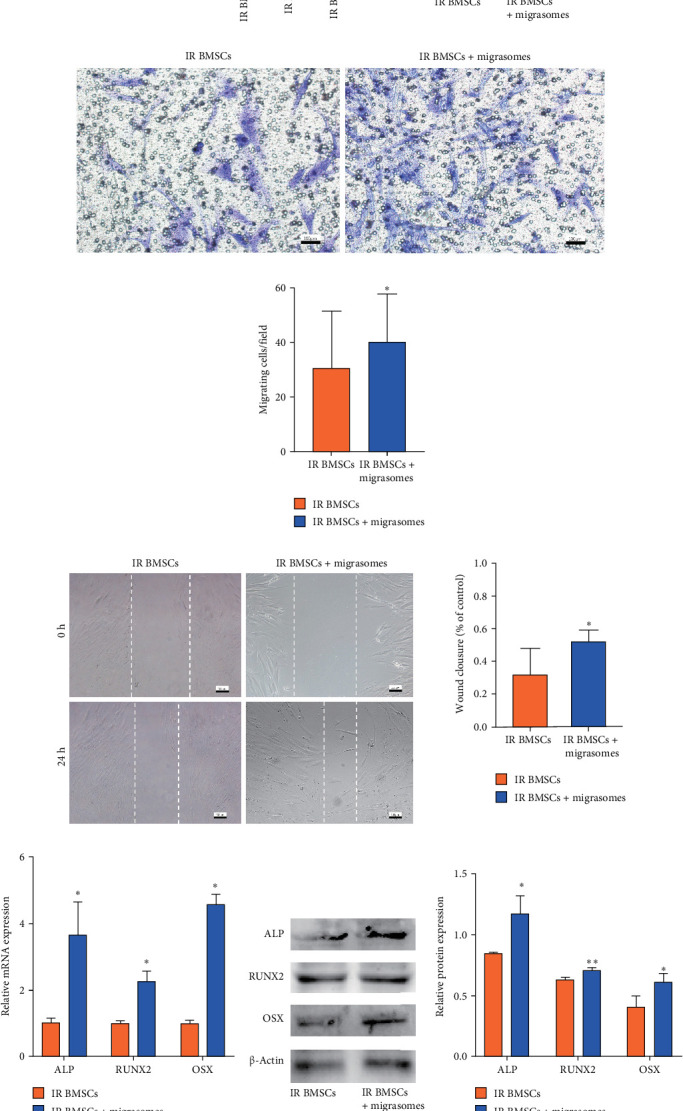
Migrasomes originating from BMSCs can enhance both the cell mobility and osteogenic capacity of IR BMSCs by transporting CYR61. (a) ALP activity of IR BMSC stimulated by varying concentrations of migrasomes, displayed a dose-dependent increase, with 2 µg/mL identified as the optimal concentration. (b) Addition of migrasomes could compensate for the CYR61 deficiency in IR BMSCs. (c) Transwell assay showed the upregulated migratory ability. (d) Quantitative analysis of transwell assay. (e) Wound healing assay further illustrated the increased migration ability induced by migrasomes. (f) Quantitative analysis based on relative RNA expression levels of *ALP*, *OSX*, and *RUNX2*. (g) Western blot analysis and statistical evaluation of migrasomes-mediated expression levels of ALP, OSX, and RUNX2 when compared to IR BMSCs alone. (h) Result of ALP staining. (i) ARS staining results. Scalar bar = 100 µm. Data are represented as mean ± SEM, *n* = 3, *⁣*^*∗∗*^*p* < 0.01, *⁣*^*∗*^*p* < 0.05.

To identify the optimal concentration for inducing IR BMSCs, the ALP activity assay was repeated and the optimal concentration was found to be 2 µg/mL (Figure 6a). We also observed that complementing IR BMSCs with migrasomes led to an upregulation of CYR61 levels (Figure 6b). Following incubation, after treating IR BMSCs with 2 µg/mL migrasomes in basal medium for 24 h, we noted a tendency for improved migration, as evidenced by increased cell migration and wound closure (Figures 6c–e). Additionally, there was an upregulation in the gene expression of osteogenic markers *ALP*, *OSX*, and *RUNX2* (Figure 6f), with a corresponding rise in protein levels observed in the migrasome-treated IR BMSCs (Figures 6g). There was also an increase in the number of cells positive for ALP staining within the group that received migrasome treatment (Figure 6h) and enhanced mineralization was evident from ARS staining (Figure 6i). These findings suggest that migrasomes have the potential to support bone regeneration and repair to a certain extent.

## 4. Discussion

This study addresses the challenges associated with ORNJ-related bone defects, which are often complicated by radiation effects on BMSCs [1, 35]. Radiation-exposed BMSCs serve as a well-established model to study cellular-level radiation injuries [5]. Irradiation at doses >2 Gy inhibited rat BMSC proliferation [10]. Specifically, a sharp decline in proliferation was observed at 7 days postirradiation, with no significant recovery evident at 3 and 14 days (2 Gy) [7]. We observed that even a 2 Gy dose can significantly influence the human alveolar-origin BMSCs' migration and osteogenesis. However, a higher dose of 4 and 6 Gy radiation was observed to alter migration and osteogenesis with cell vitality inhibited [5, 17, 36]. Importantly, our findings indicate that a radiation dose of 2 Gy does not inhibit BMSCs' vitality. Despite exhibiting reduced migratory and osteogenic capacity following 2 Gy irradiation, existing literature and our preliminary data suggest that IR BMSCs, upon appropriate modification, may hold therapeutic potential for bone regeneration in ORNJ.

In osteocytes, fluid shear stress induces CYR61, which potentially mediate mechanotransduction, thereby promoting osteoblast differentiation and bone formation. Moreover, CCN family members' expression significantly alters the cellular micro environment. These proteins exert their diverse regulatory functions—including effects on migration and differentiation—primarily by binding various integrin receptors on cell surfaces [37]. Utilizing proteomics to identify CYR61, we investigated and extracted that it can enhance the migratory and osteogenic capacities of BMSCs. Migration and differentiation are among the six core cellular processes regulated by the ERK signaling pathway [38]. CYR61 enhances osteogenic differentiation of MC3T3-E1 through the integrin αvβ3/integrin-linked kinase (ILK)/ERK signaling pathway pathway [29]. We tested for a direct protein–protein interaction between CYR61 and integrin αvβ3 using co-IP.

CYR61 also inhibits osteoclast differentiation of bone marrow macrophages through the MAPK/ERK signaling pathway [39]. Activation of ERK signaling has also been detected in short wave-induced migration of MSCs to bone defect sites, thereby accelerating bone formation and healing [40]. Our findings (CYR61-knockdown and rescue assay both) suggest that the ERK signaling pathway regulates the CYR61-mediated enhancement in migration and osteoblastic differentiation, based on both CYR61-knockdown and rescue assays.

Contrasting with its tumor-promoting role in melanoma, CYR61 drives cell migration and tumor progression [41]. CYR61-knockout mice exhibit embryonic lethality [42]. Belonging to the CCN family—a group of six structurally conserved multifunctional proteins, CYR61 shares modular domains including an insulin-like growth factor-binding protein (IGFBP) motif, a von Willebrand factor type C (vWFC) domain, thrombospondin type 1 repeats (TSP type-1) domain, heparin-binding region, and a C-terminal cysteine-knot (CTCK) domain [43]. Functionally, CYR61 operates as a matricellular growth factor that serves as an integrin ligand and incorporates into the extracellular matrix through its N-terminal heparin-binding domain [33].

Moreover, CYR61's interaction with integrin αvβ3 at the 125th aspartic acid is pivotal in human [44]. CYR61 orchestrates intestinal stem cell proliferation and differentiation via distinct signaling cascades triggered upon binding to integrin αvβ3 [45]. Our analysis included the structural visualization of the potential binding site of CYR61 and examination of sequence conservation across different species.

Furthermore, among the top differentially expressed proteins in our proteomics study, Cathepsin K, involved in osteoclastic resorption and bone remodeling disorders, was identified as the 20th most upregulated protein and IQ motif containing GTPase activating protein 3 (IQGAP3; ranked 1^st^) among the most downregulated proteins. Cathepsin K is involved in osteoclastic resorption, and considered a potential target for osteoporosis treatment [46]. Additionally, fibronectin, a key component of the ECM, is recognized for its vital role in facilitating migration [47]. Similarly, GO analysis highlighted the significance of migratory fibronectin binding and its structural role within the ECM. The restricted scope and research of these molecular analysis is one of our limitations.

The regenerative properties of MSCs and their organelles, specifically migrasomes, are promising for healing ORNJ-related defects due to their bone regeneration capabilities [31, 48]. Additionally, BMSC-derived migrasomes have demonstrated anti-inflammatory effects in poststroke pneumonia and are linked to improved migration and osteogenesis in this context [22]. This study represents the first report to document the effects of migrasomes from human alveolar-origin BMSCs in enhancing cell migration and bone formation. Migrasomes, an EVs-like organelle recently identified, are effective in restoring inhibited cellular migration when applied directly [19]. Thus, we sought to confirm the presence of CYR61 in these migrasomes and validate migrasomes' role in promoting migration and osteogenesis in IR BMSCs. Initial observations were made using WGA-488 staining and visualized through laser confocal microscopy, focusing on migrasomes emerging from BMSCs and their responsive factors [49]. Migrasomes were isolated using a combination of DC, RF, and UF, revealing structures resembling pomegranates [32]. Additionally, we identified migrasome-associated proteins TSPAN4 and EOGT on the vesicle membranes [25]. We employed a gradient concentration method to determine the optimal migrasome dosage for inducing osteogenic and migratory responses in IR BMSCs, using methods from our previous studies [50]. Our findings demonstrated that migrasome supplementation significantly increased osteoblastic differentiation and migration in IR BMSCs, correlating with elevated CYR61 levels. Intriguingly, CYR61, when encapsulated within migrasomes, was identified in the ECM where it functions as an integrin ligand with an affinity for the α subunit [51]. After treating cells with 2 µg/mL of migrasomes for 24 h, enhanced migratory capabilities were observed in wound healing and transwell assays. The osteogenic response was further confirmed by significant increases in the gene and protein expression of osteogenic markers ALP, OSX, and RUNX2, along with deeper ALP staining and increased mineralized nodules as shown by ARS staining. In summary, the migrasomes derived from BMSCs containing CYR61 were effectively transferred to IR BMSCs, restoring their diminished migratory and osteogenic abilities. The presence of CYR61 in these migrasomes were crucial in boosting their functionality, thereby highlighting the potential use of BMSC-derived migrasomes in animal models and eventual clinical applications.

Overall, our study requires further validation to confirm the function of CYR61 in vivo and to establish the critical role of migrasomes-origin CYR61 in bone regeneration under ORNJ conditions. It is essential to develop convenient and widely-used methods for the isolation of migrasomes that meet clinical needs in terms of yield, purity, and reproducibility. Additionally, considering the complex intercellular communication and micro environment, migrasomes-based approaches are still challenging [52].

## 5. Conclusions

IR BMSCs' depressed migratory and osteogenic functions are potential etiologic factors for ORNJ and associated bone defects. Deficiency of CYR61 plays a pivotal role in these abnormal functions caused by radiotherapy and can be transferred by migrasomes. Our study highlights the potential application of migrasomes-origin CYR61 in future clinical settings for applications of BMSCs in cell therapy and regenerative medicine pointing to ORNJ-related bone defects.

## Figures and Tables

**Table 1:** Primer sequences of various genes used for qRT-PCR analysis.

Gene	Sequence
*β-Actin*	F CATGTACGTTGCTATCCAGGC
	R CTCCTTAATGTCACGCACGAT
*ALP*	F CCAAAGGCTTCTTCTTGCTG
	R CCACCAAATGTGAAGACGTG
*RUNX2*	F TGGTTACTGTCATGGCGGGTA
	R TCTCAGATCGTTGAACCTTGCTA
*OSX*	F CTGACCTGCCCTATTTGTCTG
	R GCACAGTGTGATACTAGGATGC
*CYR61*	F GAGCACATGTTACTGCTTCA
	R GATAGCTGCCTCTCACAGAC

## Data Availability

The data that support the findings of this study are available from the corresponding author upon reasonable request.

## Nomenclature

ORNJ: Osteoradionecrosis of the jaw
BMSCs: Bone marrow mesenchymal stem cells
IR BMSCs: Irradiated BMSCs
RFs: Retraction fibers
TEM: Transmission electron microscope
ALP: Alkaline phosphatase
ARS: Alizarin red S
OSX: Osterix
RUNX2: Runt-related transcription factor-2
TMT: Tandem-mass-tag
TSPAN4: Tetraspanin 4
WGA: Wheat germ agglutinin
CYR61: Cysteine-rich angiogenic inducer 61
CCN: Cellular communication network
ILK: Integrin-linked kinase
ECM: Extracellular matrix.

## Supporting Information

Supporting Information Figure S1. Supporting data for Figure 4. (a) Protein interaction network identifying potential downstream targets of CYR61. (b) Conservation of amino acid sequence surrounding the 125th aspartic acid of CYR61 across various species. (c) Phosphorylation level of ERK signaling under increasing CYR61 concentration. (d) Inhibitory effect of 10 µM U0126 on ERK phosphorylation at indicated time points. Results from (c) and (d) provided the experimental basis for subsequent rescue assays. *n* = 3.
