# Supplementary figures and images for: ECM Protein CYR61 Promotes Migration and Osteoblastic Differentiation of Irradiation BMSCs via Migrasomes
Source: Stem Cells Int. 2025 Sep 21;2025:8825935. doi: 10.1155/sci/8825935 (PMC12476934; doi:10.1155/sci/8825935)

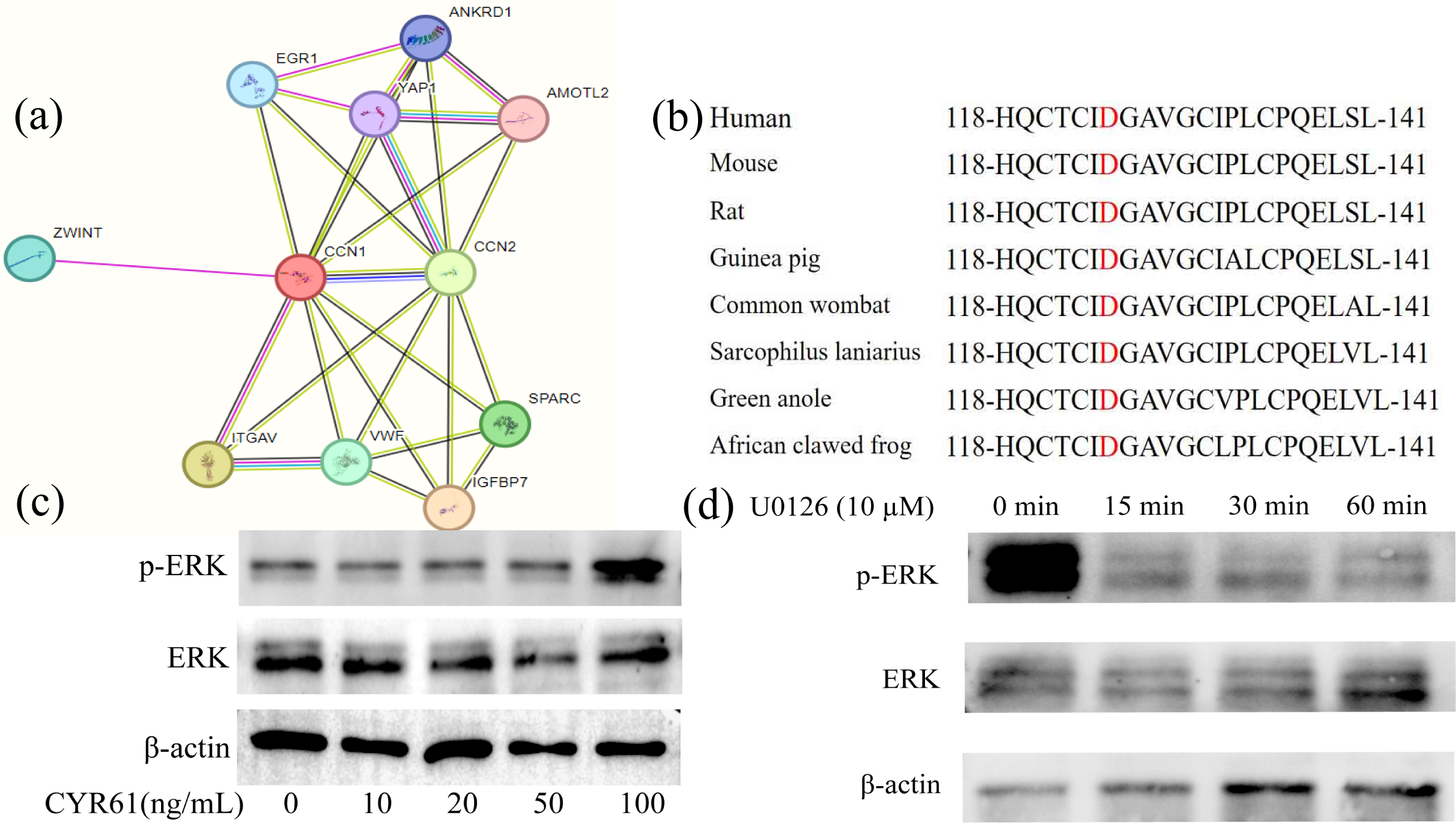

Supplement: Supporting Information — Figure S1. Supporting data for Figure 4. (a) Protein interaction network identifying potential downstream targets of CYR61. (b) Conservation of amino acid sequence surrounding the 125th aspartic acid of CYR61 across various species. (c) Phosphorylation level of ERK signaling under increasing CYR61 concentration. (d) Inhibitory effect of 10 µM U0126 on ERK phosphorylation at indicated time points. Results from (c) and (d) provided the experimental basis for subsequent rescue assays. n = 3. [file 8825935.f1.tif]
